# Structural and Predicted Functional Diversities of Bacterial Microbiome in Response to Sewage Sludge Amendment in Coastal Mudflat Soil

**DOI:** 10.3390/biology10121302

**Published:** 2021-12-09

**Authors:** Yunlong Li, Yimin Wang, Chao Shen, Lu Xu, Siqiang Yi, Yilin Zhao, Wengang Zuo, Chuanhui Gu, Yuhua Shan, Yanchao Bai

**Affiliations:** 1 College of Environmental Science and Engineering, Yangzhou University, Yangzhou 225127, China; liyunlong@yzu.edu.cn (Y.L.); wym1197653089@163.com (Y.W.); sc19971291@163.com (C.S.); xl15196239248@163.com (L.X.); ysq994382184@163.com (S.Y.); zyl18752736989@163.com (Y.Z.); 007615@yzu.edu.cn (W.Z.); shanyh@yzu.edu.cn (Y.S.); 2 Environmental Research Center, Duke Kunshan University, Kunshan 215316, China; cg294@duke.edu; 3 Jiangsu Collaborative Innovation Center for Solid Organic Waste Resource Utilization, Nanjing 210095, China

**Keywords:** coastal mudflat, saline–sodic soil, sewage sludge, microbial diversity, bacterial community, core and unique microbiomes, bacterial functional diversity

## Abstract

**Simple Summary:**

Improving microbial community and functional diversity is essential for the fertility activation and development of coastal mudflat soils, an important reserve resource for sustainable agricultural development. Although sewage sludge has been proven to be an effective agricultural practice to alleviate saline sodic stress and nutrient deficiency and thereby enable coastal mudflat reclamation, knowledge regarding the structural and functional diversities of bacterial microbiome in response to sewage sludge application remains unclear. In this study, we simultaneously investigated the effects of sewage sludge amendment on physicochemical characteristics, composition, diversity, and predicted function of bacterial community in coastal mudflat soil. Results revealed that both structural and functional diversities of bacterial microbiome were significantly improved due to microhabitat modification (i.e., mitigated saline–alkali and nutrient deficiency conditions) induced by sewage sludge amendment compared to untreated soil. Additionally, bacterial groups involved in cycling processes of carbon and nitrogen were significantly enriched in sewage sludge-amended mudflat soils. The current study enhanced our understanding of mechanisms underlying microbial community and functional diversities promoted by sewage sludge amendments.

**Abstract:**

The study investigated the influence of sewage sludge application at rates of 0 (CK), 30 (ST), 75 (MT), and 150 (HT) t ha^−1^ to mudflats on bacterial community diversity and predicted functions using amplicon-based sequencing. Soils under sewage sludge treatments, especially the HT treatment, exhibited lower pH, salinity and higher nutrient contents (C, N, and P). Moreover, restructured bacterial communities with significantly higher diversities and distinct core and unique microbiomes were observed in all sewage sludge-amended soils as compared to the control. Specifically, core bacterial families, such as *Hyphomicrobiaceae*, *Cytophagaceae*, *Pirellulaceae Microbacteriaceae*, and *Phyllobacteriaceae*, were significantly enriched in sewage sludge-amended soils. In addition, sewage sludge amendment significantly improved predicted functional diversities of core microbiomes, with significantly higher accumulative relative abundances of functions related to carbon and nitrogen cycling processes compared to the unamended treatment. Correlation analyses showed that modified soil physicochemical properties were conducive for the improvement of diversities of bacterial communities and predicted functionalities. These outcomes demonstrated that sewage sludge amendment not only alleviated saline–sodic and nutrient deficiency conditions, but also restructured bacterial communities with higher diversities and versatile functions, which may be particularly important for the fertility formation and development of mudflat soils.

## 1. Introduction

As a result of rapid population growth and urban sprawl, sustainable development of agriculture is facing serious challenges from decreasing available farmland [1,2]. The coastal mudflat, which is widely distributed worldwide, has been considered a promising alternative reserve for cultivated land resources to mitigate the issue described above [3,4,5]. Especially in China, approximately 13,000 km^2^ of coastal mudflats, more than half of the national total, have been reclaimed in the recent decades to meet the ever-growing land resource demands for industrial and agricultural development [6,7]. However, due to long-term seawater immersion and intensive evapotranspiration, coastal mudflat soils are characterized by high salinity, sodicity, and nutrition deficiency, which seriously influence the newly reclaimed soil’s health and subsequent cultivation performance [8,9,10,11]. Consequently, the development of effective and efficient agricultural practices is of significance for overcoming these obstacles in exploiting mudflat soils or other saline–sodic soils [12,13].

Alleviating saline–alkali stress and increasing organic matter have been considered as significant prerequisites for successful sodic and saline–sodic soils amelioration [14]. Currently, agricultural practices ameliorating coastal saline–alkali soils mainly include physical, biological, and chemical regimes [1,15]. As to physical methods, leaching, irrigation, and drainage can reduce soil salt content and pH [12]. For biological approaches, breeding and cultivating crops with high salt tolerance have also been proposed as an alternative strategy for soil conditioning in the coastal zone [16,17]. However, these methods are expensive and time-consuming practices that severely restrict their wide application across the world. In terms of chemical methods, organic amendments (e.g., farmyard and poultry manures, sewage sludge, compost, biochar, and polyacrylamide) have positive influences on saline–sodic soils quality enhancement and crop yield improvement demonstrated by numerous studies [1,18,19,20]. In particular, sewage sludge, a byproduct of wastewater-treatment system, has been increasingly considered an economically feasible soil conditioner because they are abundant and rich in nutrients such as phosphorus, nitrogen, and carbon [21,22]. Furthermore, sewage sludge has been confirmed as an effective practice to reduce salinity and pH, enhance nutritional status, accelerate water-stable aggregate formation and stability, and promote crop productivity in coastal mudflat soils [14,20,23,24].

Besides physicochemical properties, microbial community and functional diversities are also recognized as particularly important regulators involved in soil remediation [25,26]. Moreover, studies have shown close correlations between soil physicochemical properties and diversities of microbial community and function [27]. Organic amendments improve soil abiotic stresses for microbial colonization; in turn, soil microorganisms are important participants in the cycling processes of nutrients such as carbon, nitrogen, and phosphorus, especially in coastal regions [28,29]. Therefore, it is of great significance to comprehensively understand the responses of these environmental factors, microbiomes, and their community functions in coastal mudflat soils to the application of sewage sludge. Several studies on the influence of sewage sludge application on the soil attributes have found that (i) sewage sludge application enabled the restructuration of soil microbiome, resulting in shifts in microbial composition and structure [30,31], (ii) the organic amendment had enormous impacts on microbial biomass, microbial activity (i.e., basal respiration rate and metabolic quotient), enzymatic activity, and C- or N-related cycling [32,33], (iii) diversities of microbial community and function mainly correlated to the principle environmental factors such as pH, salinity, and nutrients [34,35]. Nevertheless, contrasting results have also been observed due to the multiple soil types, sludge types, reclamation time, application methods, and additive amounts [27,36,37]. However, up to now, most of these findings described above were observed in agricultural lands and knowledge regarding the responses of soil microbiomes and functions in coastal mudflat soils to sewage sludge amendment is still rudimentary.

Some recent studies observed the shifts of microbial communities and biological activities in response to exogenous none-sludge organic amendments in coastal saline soils. For instance, Jiang et al. [15] and Yao et al. [38] found that bio-organic amendment incorporated with chemical amendment significantly increased bacterial absolute abundance and diversities in coastal saline soils. Xie et al. [39] also reported that exogenous organic amendments (i.e., chicken manure incorporated with straw, straw mulching, and polyacrylamide, etc.) not only had a strong impact on soil physicochemical attributes, but also significantly enhanced multiple enzymatic activities in newly reclaimed tidal mudflats. According to these findings, we hypothesized that sewage sludge amendment might be capable of reassembling bacterial microbiomes and altering relevant functions. In this study, field experiments were conducted in mudflat soils to investigate the influences of sewage sludge at rates of 0, 30, 75, and 150 t ha^−1^ on soil physicochemical factors, bacterial microbiomes, and functional potentials. The specific objectives of this study were to: (i) reveal bacterial microbiome alterations in response to sewage sludge amendment, (ii) evaluate the influences of sewage sludge application on soil putative functions, especially in carbon and nitrogen cycling, (iii) determine the mechanisms that explain the shifts of soil bacterial community and functionality induced by sewage sludge. 

## 2. Materials and Methods

### 2.1. Study Site and Sewage Sludge

The field experimental area was situated in Rudong County (32°20′ N, 121°23′ E), Jiangsu Province, southeast China, located at an approximate distance of 1.0 km from the Huanghai Sea coastline. The altitude of this area is about 4 m above sea level, with a subtropical oceanic monsoon climate zone. The mean annual temperature is 15 °C, and mean annual rainfall is 1000 mm. Soil at the experimental area is typical saline–alkali soil and classified as the Aalaquepts group of Aquepts in Inceptisols based on USDA classification systems. Main physicochemical properties of mudflat soil used in the current study were listed in Table S1.

Sewage sludge used in the present study was produced by the Rudong municipal wastewater treatment plant in Jiangsu Province (China) and met the requirements which are specified in the Chinese national standards. The main physicochemical characteristics of sewage sludge and corresponding Chinese national standards are listed in Table S1.

### 2.2. Experimental Design, and Soil Sampling

To achieve the average content of organic matter in agricultural soil (0.5~1.5%), sewage sludge was applied once into the soil at rates of 0 (CK), 30 (ST), 75 (MT), and 150 (HT) t ha^−1^ on a dry weight basis and incorporated thoroughly into topsoil using a rotavator. The four treatments were set up in a randomized block design, and each treatment contains three replications (plots) with each plot being 16 m^2^ (4.0 m × 4.0 m). Three salt-enduring crops, namely Ryegrass (*Lolium perenne* L.), Maize (*Zea mays* L.), and Sesbania (*Sesbania cannabina*), were consecutively cultivated and rotated from 2011 to 2014. All plants were supplied with rainwater and neither irrigation nor fertilization were applied during the whole experiment. After the 3-year crop cultivation, soils were sampled randomly at a 0 to 20 cm depth from each replicate for all treatments at the end of September 2014. Each soil sample was a composite of five soil cores. Collected soil samples were ground, sieved (2 mm mesh), and stored as appropriate for subsequent physicochemical and biological analysis. 

### 2.3. Soil Physicochemical Assays

Soil physicochemical properties, including pH, salinity, total organic carbon (TOC), nitrogen (TN), and phosphorus (TP), alkaline N (AN), and available P (AP), were measured in this study according to Lu [40]. Briefly, soil pH was determined in 1:5 (mass/volume) soil/water extracts using a pH meter. Soil salinity was determined using the gravimetric method. TOC, TN, and TP of soil samples were determined using K_2_Cr_2_O_7_-H_2_SO_4_ digestion, Kjeldahl digestion, and Mo-Sb colorimetry methods, respectively. Alkaline hydrolysis diffusion and molybdenum blue methods were used to measure the soil AN and AP, respectively.

### 2.4. DNA Extraction and 16S rDNA Amplicon Sequencing 

Total genomic DNA was extracted from soil samples (500 mg) using a FastDNA^®^ SPIN Kit (MP Biomedicals, Santa Ana, CA, USA) following the manufacturer’s instructions, and the DNA extracts were dissolved into 100 μL elution buffer. Quality and quantity of DNA samples were determined based on the ratios of A260/A280 nm and A260/A230 nm using NanoDrop ND-1000 spectrophotometer (ThermoFisher Scientific, Waltham, MA, USA). Finally, DNA samples were preserved at −80 °C for subsequent Miseq sequencing. 

For PCR amplification, the primers targeting the V4 region of bacterial 16S rDNA were 515-F (5′-GTG CCA GCM GCC GCG GTA A-3′) and 806-R (5′-GGA CTA CHV GGG TWT CTA AT-3′). The PCR reactions used in this study were according to the established method detailed in the previous report [41]. PCR products were pooled in equimolar and then purified using AMpure XP beads. Paired-end sequencing was performed on an Illumina Miseq instrument at Huada Biotechnologies, Inc. (Wuhan, China) according to standard protocols. 

### 2.5. Data Processing of Bioinformatics

Software QIIME (v.1.9.1) was used to process raw sequencing data, according to the methods described by Zhao et al. [42]. Briefly, raw fastq files were combined and barcodes removed using scripts of ‘multiple_join_paired_ends.py’ and ‘multiple_extract_barcodes.py’, respectively. Then, the merged reads were quality controlled using the script of ‘multiple_split_libraries_fastq.py’. Reads were clustered into operational taxonomic units (OTUs) using the script of ‘pick_open_reference_otus.py’ at 97% sequence similarity against the Greengenes13_8 database [43]. The phylogenetic affiliation of individual OTUs was achieved based on the representative sequences using the Ribosomal Database Project (RDP) naïve Bayesian rRNA classifier with the confidence threshold of 80% [44]. Particularly, chloroplast and mitochondria OTUs were filtered from the OTU table using the script of ‘filter_otus_from_otu_table.py’. 

Rarefaction analysis at a unified depth (20,000) of each sample was conducted to reveal the alpha (α-) diversity indices including Shannon diversity, observed species (richness), and Pielou’s evenness. Principal coordinates analysis (PCoA) and hierarchical cluster analysis were performed to examine beta (β-) diversities across all samples based on Bray–Curtis distance matrices. In this study, OTUs present in all biological replicates of each treatment were selected and defined as core OTUs, and OTUs present only in the three biological replicates of one treatment were defined as unique OTUs. Additionally, Functional Annotation of Prokaryotic Taxa (FAPROTAX) was adopted to further predict the potential metabolic and ecological functions of core and unique OTUs across bacterial communities in all treatments [45]. The most updated FAPROTAX (version 1.2.1) was used in the present study. The predictions were obtained from core and unique OTUs tables annotated against a database containing 90 functional groups with 8236 members. 

### 2.6. Statistical Analysis

One-way ANOVA followed by the Duncan post hoc test were used to analyze the differences across four treatments at the 5% confidence level in SPSS (Version 19.0, SPSS Inc., Chicago, IL, USA). A significant difference test of bacterial community structure and the functional profile of core and unique OTUs across different treatments was conducted using Permutational multivariate analysis of variance (PERMANOVA, *adonis*) based on Bray–Curtis distance matrices [46]. Post hoc multiple comparisons were carried out to test the difference between treatments using pairwise Adonis with FDR correction in R version 4.0.2. In addition, permutational analysis of multivariate dispersions (PERMDISP) were performed to verify the PERMANOVA results. Heatmaps of taxa and function abundances were generated based on Z-score transformed values of relative abundances of bacterial phyla, families, and predicted functions in different treatments. The complete linkage clustering method was used for clustering in heatmaps. Linear Discriminant Analysis Effect Size (LEfSe) was used to further elucidate the biomarkers in the bacterial communities among soils amended by sewage sludge and non-amended soils [47]. In LEfSe analysis, differences were considered significant with the logarithmic LDA score > 4 and *p* < 0.05 (Kruskal–Wallis sum-rank test). Spearman’s rank correlations were conducted to examine the relationships between soil physicochemical characteristics and the diversities of bacterial community and putative functions as well as between core microbiomes and carbon- and nitrogen-related functional categories.

## 3. Results

### 3.1. Influences of Sewage Sludge on Soil Physicochemical Properties

Overall, lower pH and salinity and higher content of nutrients (C, N, and P) were observed in sewage sludge-treated soils (Table 1). In particular, there were no significant differences in soil pH and salinity among CK, ST, and MT treatments. In contrast, values of soil pH and salinity in HT were significantly (*p* < 0.05) lower than those in CK. TOC, TP, AN, and AP in sewage sludge-amended soils were significantly (*p* < 0.05) higher than those in CK treatment, with the highest value observed in HT and the lowest in ST treatment. Additionally, the total nitrogen content in both HT (1.38) and MT (1.08) soils was significantly (*p* < 0.05) higher than that in CK (0.47).

### 3.2. α- and β-Diversity Analysis of Bacterial Community

**Figure 1:**
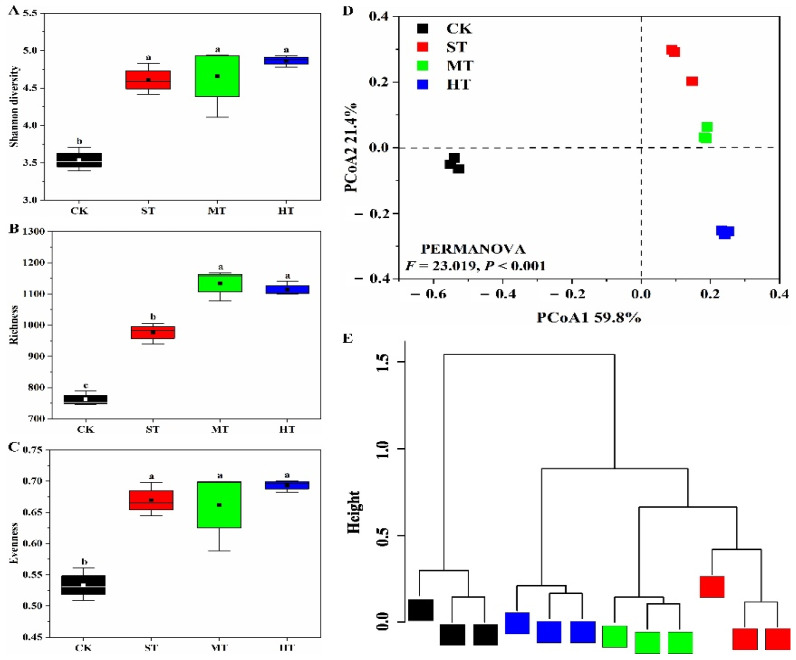
Bacterial community structure and diversity in coastal mudflat soils with different sewage sludge application rates. (**A**–**C**) represent Shannon diversity, richness, and evenness of bacterial communities, respectively; (**D**,**E**) indicate principal coordinate analysis and hierarchical cluster analysis of bacterial communities based on the Bray–Curtis distance metric. Error bars indicate the standard errors of the means of three replicates. Different letters represent significantly different values at *p* < 0.05 according to Duncan’s multiple range test. Treatments: CK, ST, MT, and HT indicate 0, 30, 75, and 150 t ha^−1^ sewage sludge applied in mudflat saline soils, respectively.

In general, alpha diversity indices (Shannon diversity, richness, and evenness) of soil bacterial communities in sewage sludge treatments were significantly (*p* < 0.05) higher than those in CK (Figure 1). However, there were no significant differences in Shannon diversity and evenness of bacterial communities among sewage sludge-amended soils (Figure 1A,C). Richness in MT and HT soils was significantly higher (*p* < 0.05) than in the ST group. However, there were no significant differences in richness of bacterial communities between MT and HT (Figure 1B). 

Sewage sludge application significantly (PERMANOVA, *p* < 0.001; PERMDISP, *p* = 0.793) altered soil bacterial community structure (Figure 1D and Table S2). The first and second principle components of PCoA ordination plot expressed 59.8% and 21.4% of the overall variance across the four treatments, respectively. Soil samples of ST, MT, and HT harbored distinct bacterial community structures as compared to CK. In particular, significant (pairwise PERMANOVA, *p* < 0.05) differences were found in terms of bacterial community structures between CK and HT soils. Hierarchical cluster analysis showed that bacterial community structures among the four treatments formed two distinct clusters (Figure 1E). Particularly, bacterial community structures in soils amended by sewage sludge were similar and together formed a distinct cluster, separating from the other cluster grouped by CK treatment. In addition, the bacterial community structure in ST was similar to MT, which was separated from HT treatment.

### 3.3. Soil Bacterial Community Composition

**Figure 2:**
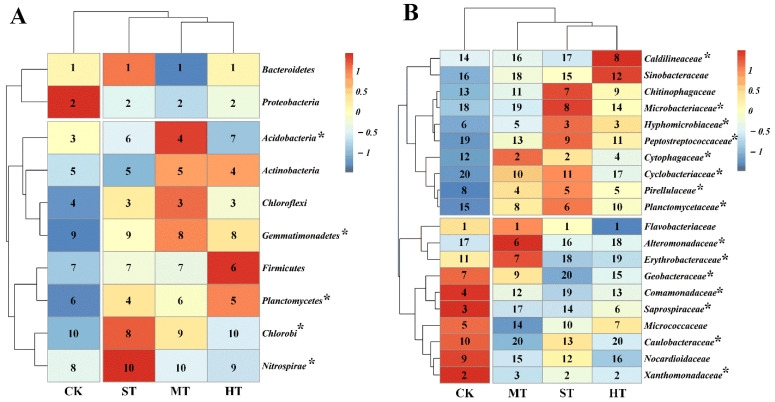
Taxonomic differences in bacterial community at multiple levels. Heatmap displaying the relative abundance of the abundant bacterial phyla (top 10) (**A**) and family (top 20) (**B**) across treatments. The key from blue to red represents the least abundant to most abundant for given taxa across four treatments. Numbers from 1 to 20 in same column represent ranks of given phyla or family within a single treatment. Asterisks (*) at the upper right of taxon indicate *p* < 0.05 according to Duncan’s multiple range test. Treatments: CK, ST, MT, and HT indicate 0, 30, 75, and 150 t ha^−1^ sewage sludge applied in mudflat saline soils, respectively.

Sewage sludge application considerably modulated the bacterial community from genus to phylum level (Figure 2 and Figure S1; Table S3). Specifically, the heatmap indicated that bacterial community composition of ST, MT, and HT grouped and separated from CK. Relative abundances of *Acidobacteria*, followed by *Planctomycetes*, *Gemmatimonadetes*, *Nitrospirae*, and *Chlorobi* were significantly (*p* < 0.05) altered as a result of sewage sludge application (Figure 2A). Similarly, the cluster results at family level were in accordance with the phylum level (Figure 2B). *Flavobacteriaceae* families, followed by *Xanthomonadaceae* and *Hyphomicrobiaceae* families were predominant families across different treatments, with average relative abundances of 36.2%, 9.6%, and 3.8%, respectively. Particularly, compared with CK, relative abundances of *Hyphomicrobiaceae* were significantly (*p* < 0.05) higher in soils amended by sewage sludge. In contrast, significantly (*p* < 0.05) lower relative abundances of *Xanthomonadaceae* were observed in sewage sludge groups when compared to that in CK (Figure 2B and Table S3).

LEfSe analysis showed that sewage sludge considerably altered bacterial community compositions in different treatments at multilevel taxa (Figure S1). Overall, 38 biomarkers were identified among different soil samples, with 12, 8, 4, and 14 biomarkers associated with the CK, ST, MT, and HT soils, respectively. In particular, *Gillisia* was significantly (LDA = 5.13, *p* = 0.02) abundant in CK treatment. While *Cytophagia* (LDA = 4.50, *p* = 0.02), *Acidobacteria* (LDA = 4.59, *p* = 0.02), and *Arenibacter* (LDA = 5.08, *p* = 0.02) were significantly enriched in ST, MT, and HT soils, respectively.

### 3.4. Core and Unique Bacterial Microbiomes

**Figure 3:**
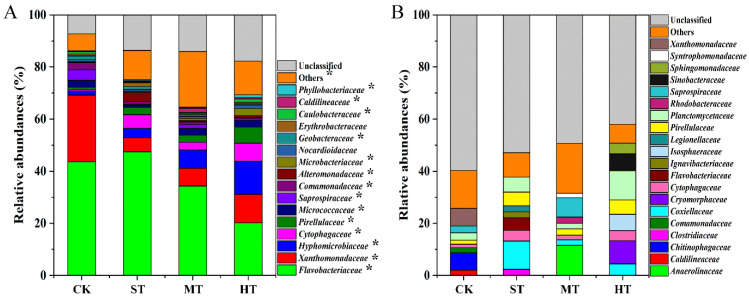
Relative abundances of bacterial families in core OTUs (**A**) and unique OTUs (**B**) in soils with different sewage sludge application rates. Only families with relative abundances higher than 1% in core OTUs and top-ten families in unique OTUs are presented. Asterisks (*) at the upper right of taxon in core OTUs indicate *p* < 0.05 according to the Duncan’s multiple range test. Treatments: CK, ST, MT, and HT indicate 0, 30, 75, and 150 t ha^−1^ sewage sludge applied in mudflat saline soils, respectively.

Distinguishable core and unique bacterial microbiomes were observed in soils amended by sewage sludge as compared to CK (Figure 3). Number of core OTUs in all treatments was 215, accounting for 18.1% of the total selected OTUs (1191) (Table 2). Core OTUs in CK, ST, MT, and HT treatment accounted for 41.8, 29.9, 28.1, and 33.9% of the selected OTUs and 85.4, 72.8, 66.1, and 48.5% of the selected sequences, respectively (Table S4). The majority (64.6–86.1%) of the core OTUs were affiliated into 16 families, and the relative abundances of 14 families shifted significantly (*p* < 0.05) across different treatments (Figure 3A and Table S5). Particularly, relative abundance of *Flavobacteriaceae*, the dominant core families in all treatments, considerably (*p* < 0.05) shifted from 43.6% (CK) to 47.4% (ST), 34.3% (MT), and 20.2% (HT), respectively. Compared with CK treatment, families *Xanthomonadaceae*, *Saprospiraceae*, *Comamonadaceae*, and *Geobacteraceae* were significantly (*p* < 0.05) depleted, while families *Hyphomicrobiaceae*, *Cytophagaceae*, *Pirellulaceae*, *Alteromonadaceae*, *Microbacteriaceae*, *Caldilineaceae*, and *Phyllobacteriaceae* were significantly (*p* < 0.05) enriched in all sewage sludge-amended soils. 

The number of total unique OTUs in all treatments were 449 and accounted for 37.7% of the total selected OTUs (Table 2). In particular, numbers of OTUs unique to CK, ST, MT, and HT treatments were 106, 136, 112, and 95, respectively, accounting for 20.6, 18.9, 14.7, and 15.0% of the total selected OTUs and 2.2, 2.8, 1.6, and 1.5% of the selected sequences, respectively (Table S4). Furthermore, 22.8–37.1% of the unique OTUs in all treatments were classified into 20 families, and the numbers of the unique families in CK, ST, MT, and HT soils were 8, 8, 8, and 8, respectively (Figure 3B and Table S6). In particular, the most abundant unique families altered from *Chitinophagaceae* in CK to *Coxiellaceae* (ST), *Anaerolinaceae* (MT), and *Planctomycetaceae* (HT). (Figure 3B). It is noteworthy that species belong to families *Caldilineaceae*, *Chitinophagaceae*, *Comamonadaceae*, and *Xanthomonadaceae* only appeared in CK treatment, while species belonging to families *Coxiellaceae* were only observed in sewage sludge-amended soils. In addition, species of *Clostridiaceae*, *Flavobacteriaceae*, *Ignavibacteriaceae*, and *Legionellaceae* were only found in ST group. Species affiliated to *Anaerolinaceae*, *Rhodobacteraceae*, and *Syntrophomonadaceae* were only enriched in MT group. Species assigned to *Cryomorphaceae*, *Isosphaeraceae*, *Sinobacteraceae*, and *Sphingomonadaceae* only appeared in HT soil (Table S6).

### 3.5. Functional Prediction of Core and Unique OTUs

**Figure 4:**
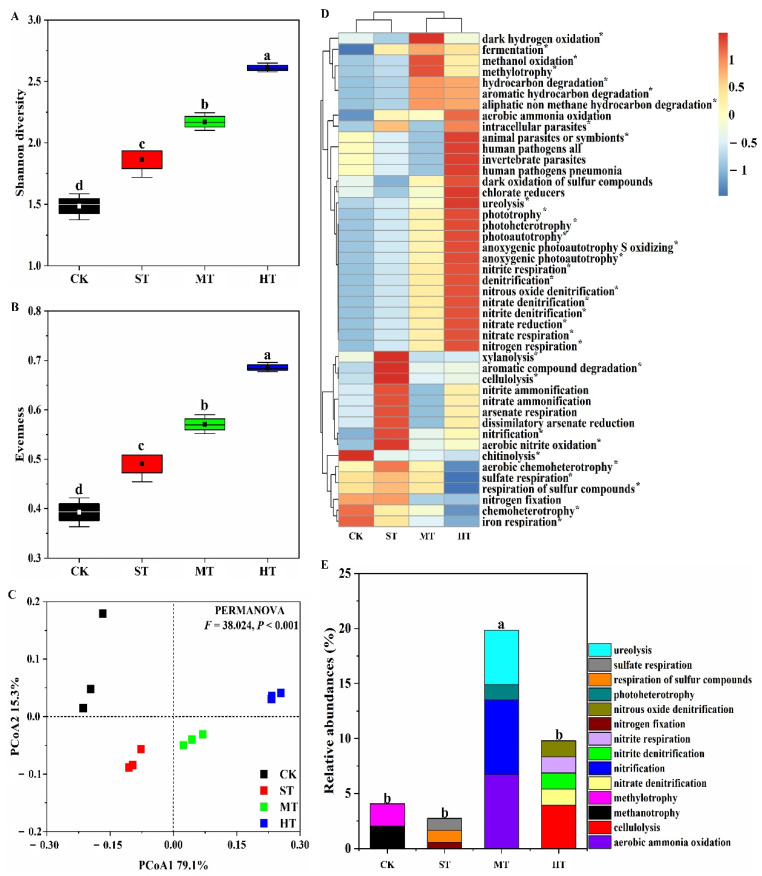
Metabolic and ecological functions of core and unique OTUs in soils under different treatments based on FAPROTAX. (**A**,**B**) represent Shannon diversity and evenness of the predicted functions of core OTUs, respectively; (**C**) indicates PCoA of functional community structure of core OTUs; (**D**) function profiles of core OTUs across different treatments; the key from blue to red represents the least abundant to most abundant in each row for a given function; (**E**) changes in relative abundances of C- and N-related functions of unique OTUs in soils under different treatments. Different letters and asterisks (*) indicate *p* < 0.05 according to Duncan’s multiple range test. Treatments: CK, ST, MT, and HT indicate 0, 30, 75, and 150 t ha^−1^ sewage sludge applied in mudflat saline soils, respectively.

Sewage sludge application significantly (*p* < 0.05) altered functional profiles of bacterial core microbiome. The HT treatment exhibited a distinct influence when compared to ST and MT treatments (Figure 4). In general, sewage sludge increased alpha diversities of the functional traits with significantly (*p* < 0.05) higher Shannon diversity and evenness observed in ST, MT, and HT treatments as compared to CK treatment (Figure 4A,B). Furthermore, sewage sludge-amended soils harbored significantly (PERMANOVA, *p* < 0.001; PERMDISP, *p* = 0.277) distinct functional community structures, which together separated from CK according to the principal coordinate analysis as well as functional clustering results (Figure 4C). In particular, significant (pairwise PERMANOVA, *p* < 0.05) differences were observed in terms of functional community structures between CK and HT treatment (Table S2). 

Overall, 29.8% of core OTUs, 16.2%, 25.0%, 14.3%, and 21.1% of unique OTUs of CK, ST, MT, and HT were assigned to at least one functional group, respectively. More functional categories with significantly higher relative abundances were observed in sewage sludge-amended soils. A total of 42 categories were linked to the bacterial core microbiomes, and significant (*p* < 0.05) variations in the relative abundance of 33 functional traits were observed across different treatments (Figure 4D). The most abundant functional traits were nitrate reduction, nitrate respiration, and nitrogen respiration, with average relative abundances of 1.9%, 1.9%, and 1.9% across different soil groups, respectively. In particular, a total of 28 functional traits associated with biogeochemical cycles of C (13) and N (15) were obtained in this study (Tables S7 and S8). In C processes, significantly (*p* < 0.05) higher accumulative relative abundances of carbon cycling processes were observed in sewage sludge-amended soils, with the highest value found in HT treatment (Table S7). Sewage sludge applications significantly (*p* < 0.05) improved functional traits involved in anoxygenic photoautotrophy S oxidizing, anoxygenic photoautotrophy, photoautotrophy, photoheterotrophy, and phototrophy. In addition, significantly (*p* < 0.05) higher relative abundances of xylanolysis, aromatic compound degradation, and cellulolysis were observed in ST treatment, and methanol oxidation and methylotrophy in the MT group were detected as compared to other treatments. Relative abundances of functional pathways involved in aromatic hydrocarbon degradation, aliphatic non-methane hydrocarbon degradation, and hydrocarbon degradation were significantly (*p* < 0.05) up-regulated both in MT and HT groups as compared to CK and ST treatments. Similarly, in N processes, significantly (*p* < 0.05) higher accumulative relative abundances of nitrogen cycling processes were observed in sewage sludge-amended soils (Table S8). Functions such as nitrate reduction, nitrate respiration, nitrogen respiration, nitrite respiration, nitrate denitrification, nitrite denitrification, nitrous oxide denitrification, and denitrification were significantly (*p* < 0.05) up-regulated in all sewage sludge-amended soils as compared to CK. Relative abundances of nitrification and aerobic nitrite oxidation in ST treatment, and ureolysis in HT treatment were significantly (*p* < 0.05) higher than other treatments.

Unique OTUs in different treatments were associated with 31 functional categories (Table S9). Numbers of these predicted functional categories in CK, ST, MT, and HT treatments were 14, 13, 14, and 14, respectively. In addition, 2, 3, 4, and 5 carbon- or nitrogen-related functions were only observed in CK, ST, MT, and HT treatments, respectively, with the significantly (*p* < 0.05) higher accumulative relative abundance observed in MT treatment (Figure 4E). Specifically, methanotrophy and methylotrophy were only detected in CK treatment. Functions related to nitrogen fixation, respiration of sulfur compounds, and sulfate respiration were only detected in ST treatment. Functions aerobic ammonia oxidation, nitrification, photoheterotrophy, and ureolysis were only detected in MT treatment, while cellulolysis, nitrate denitrification, nitrite denitrification, nitrite respiration, and nitrous oxide denitrification were only detected in HT soil.

### 3.6. Correlations of Soil Bacterial Community and Environmental Factors

Spearman’s rank-order correlation analysis showed that soil pH significantly (*p* < 0.01) and negatively correlated with bacterial community structure (Table 3). Likewise, soil salinity exhibited a significant (*p* < 0.05) and negative correlations with Shannon diversity, evenness, and bacterial community structure. Soil nutrients, including TOC, TN, TP, AN, and AP, were significantly (*p* < 0.05) and positively related to most of α- and β-diversities of bacterial microbiome. 

**Figure 5:**
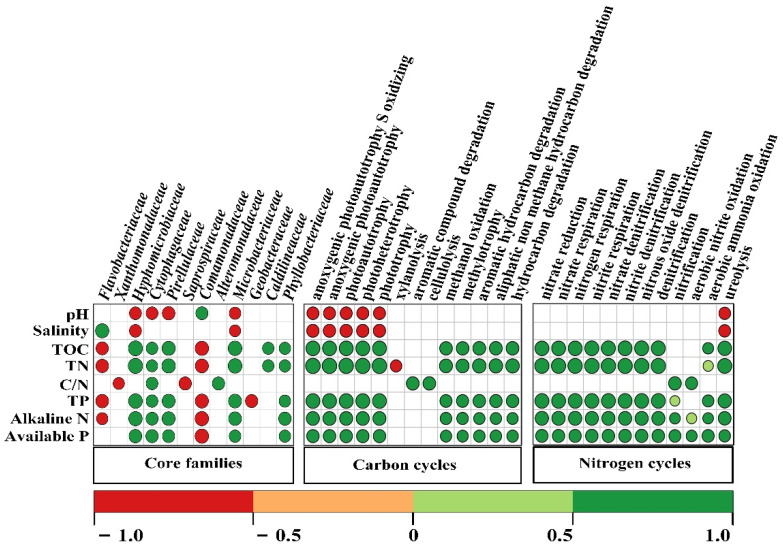
Spearman’s rank-order correlation analysis between soil physicochemical characteristics and core bacterial families, carbon, and nitrogen related functions. Only the significant correlations are presented. The circles in red and green represent negative correlation and positive correlation.

In particular, significant relationships were found between soil environmental factors and core OTUs, C- and N-associated functional categories (Figure 5). Specifically, environmental factors were found to be significantly (*p* < 0.05) related to 12 of the total classified 16 core families. Families *Hyphomicrobiaceae*, *Cytophagaceae*, *Pirellulaceae*, *Microbacteriaceae*, *Caldilineaceae*, and *Phyllobacteriaceae* were significantly enriched in soils amended by sewage sludge, exhibited significant (*p* < 0.05) and positive relationships with most soil nutrient contents. In terms of functions, a total of 13 and 12 functions associated with carbon and nitrogen processes significantly (*p* < 0.05) correlated with soil physico-chemical characteristics. Soil pH and salinity significantly (*p* < 0.05) and negatively related to ureolysis and phototrophy related functions, including anoxygenic photoautotrophy S oxidizing, anoxygenic photoautotrophy, photoautotrophy, photoheterotrophy, and phototrophy. Most of the C- and N-related biogeochemical cycles significantly (*p* < 0.05) and positively correlated with soil contents of TOC, TN, TP, AN, and AP, with the exception of xylanolysis.

## 4. Discussion

### 4.1. Sewage Sludge Significantly Alleviated Saline–Alkali Stresses and Elevated Nutrient Availability in Coastal Mudflat Soil

Increasing evidence has demonstrated that sewage sludge usage can ameliorate salt and alkaline stresses and promote nutritional status in mudflat soils [1,2]. Apparently, sewage sludge application exhibited significant influence on most of the soil physicochemical attributes in the present study (Table 1). This was consistent with our previous studies that found sewage sludge has significant effects on soil alkalinity, salinity, and nutrient retention [14,23,24,48]. Specifically, soil pH was significantly lower in sewage sludge-amended soils as compared to CK treatment (Table 1), which might be partially attributed to neutralization caused by lower-pH sewage sludge (6.32, Table S1) and organic acids (i.e., humic and fulvic acids) generated during the degradation of organic matters in sewage sludge [6,49]. Additionally, cumulative H+ secreted by plants roots and increased CO_2_, NO_3_^−^, and NO_2_^−^ levels [50,51,52,53] might also be responsible for the decline in mudflat soil pH. It is noting that, compared with ST and MT, lower soil pH was observed in HT treatment, which might be explained by more protons and organic acids accumulated in HT soil samples, as it had more organic matter for decomposition. 

Unsurprisingly, considerably increased soil organic carbon content and decreased soil salinity were observed in sewage sludge-amended treatments as compared to unamended soil (Table 1). The reason lies in the fact that applying sewage sludge with abundant organic matter into mudflat salt-affected soil can not only rapidly elevate soil organic carbon stocks [54], but also significantly facilitate water-stable aggregate formation, which efficiently prevents saline water from moving upward through soil capillaries, and finally reduced salt in the plough layer [6]. Apart from the soil organic carbon, sewage sludge application also promoted nitrogen and phosphorus contents in mudflat soil, which increased with the application rate of sewage sludge (Table 1). Consistent with previous literature, our results showed that sewage sludge with abundant nutrients is a prominent amendment for improving nutritional status in mudflat soils [48,55,56]. Overall, these findings showed that sewage sludge alleviated saline–alkali stresses and promoted nutrient storage in mudflat soil, suggesting applying sewage sludge might be a feasible and effective fertilizer regime for coastal mudflat soils fertility enhancement.

### 4.2. Sewage Sludge-Amended Coastal Mudflat Soil Harboring Diverse Bacterial Community Structure and Diversity

In addition to the amendment of soil physical and chemical properties [14,57], soil biological components, especially the soil microbial communities have been widely demonstrated to positively respond to the organic input managements [30,37]. In this study, significantly higher Shannon diversity, richness, and evenness were observed in all sewage sludge-amended soils as compared to CK treatment. (Figure 1A–C), which were in accordance with previous investigations that showed sewage sludge significantly increased the compositional diversities of bacterial communities in different types of soils [31,35,37]. One possible explanation is that alleviated saline–alkali stresses and improved nutritional status caused by sewage sludge application might facilitate more diverse microbial colonization in mudflat soils [58,59,60,61]. Additionally, divergent microbial species populated in sewage sludge were brought into mudflat soil, which might be responsible for the higher α-diversity values observed in sewage sludge treatments [30]. Besides the compositional diversity, distinct bacterial community structures in sewage sludge treatments were observed as compared to control soil, especially for HT treatment (Figure 1D,E), which was interpreted to be a result of the dosage-effect on the soil microbial communities induced by the sewage sludge with different application amounts [62]. The result was consistent with previous findings of Liu et al. [35], who also found distinctive bacterial community structures in soils amended by different amounts of composted sewage sludge. 

Decoding core and unique microbiomes is important in deciphering the consistent and distinctive responses of soil microbial communities to different treatments [63,64]. Our results showed that sewage sludge application significantly altered the entire bacterial community composition from genus to phylum level (Figure 2 and Figure S1) and, similarly, the bacterial core and unique microbiomes (Figure 3 and Table 2). At phylum level, *Bacteroidetes*, followed by *Proteobacteria*, *Chloroflexi*, *Actinobacteria*, *Acidobacteria* and *Planctomycetes*, are predominant bacterial phyla across all treatments (Figure 2A). It was in line with previous observations that showed these phyla are ubiquitous and widely distributed in the saline–alkaline environment [15,65,66,67]. In particular, *Planctomycetes* populations flourished in all sewage sludge soils (Figure 2A). The result was in agreement with Ke et al. [68], who found a considerably higher relative abundance of *Planctomycetes* in the soil after cultivation following sewage sludge application. For core bacterial communities, the largest increases relative to CK in relative abundances occurred within the families of *Hyphomicrobiaceae*, followed by *Cytophagaceae*, and *Pirellulaceae*, in sewage sludge-amended soils (Figure 3A). This result was likely because species affiliated to these families, frequently isolated from wastewater treatment plants, were introduced by sewage sludge into mudflat soil and favored the growth by the amended physicochemical properties therein. It is worth noting that abundant members belonging to the families mentioned above have been widely demonstrated to be associated with carbon substrate utilization and nitrogen related functions in soils, strongly implying the improvement of mudflat soil functional capabilities induced by sewage sludge amendment [69,70,71,72,73]. Regarding unique microbiomes, distinct profiles of bacterial communities were observed across four treatments, and the profiles were dramatically different from the soil core bacterial communities (Figure 3B), indicating that the unique microbiome in mudflat soil more relied on the sewage sludge application. In particular, the *Coxiellaceae* family, which have been widely reported to be capable of degrading multiple carbon-containing compounds and secreting plant-protective substances [74], were only observed in sewage sludge-amended soils, strongly suggesting that sewage sludge can assemble a unique multifunctional microbiome in mudflat soil. The soil bacterial microbiome is an integral component of soil ecosystem and plays pivotal role in maintaining multitudinous biogeochemical processes [26]. The present study shows that sewage sludge application was able to drive mudflat soil bacterial community assembly with higher community diversity and distinguishable core and unique microbiomes. Meanwhile, flourishment of bacterial species possessing various functional potentials might indicate the versatile functions of bacterial microbiomes in sewage sludge-amended coastal mudflat soil.

### 4.3. Sewage Sludge Application Potentially Contributed to the Predicted Functional Diversity Improvement in Mudflat Soil

Microbial community functional diversity is increasingly viewed as the most ecologically associated biodiversity indicator, all of which are crucial for comprehending the mechanisms of microbial communities in soil functions assemblage under different environmental constraints [75,76]. Notably, results in this study showed that sewage sludge application significantly promoted Shannon diversity and evenness of putative metabolic and ecological functional traits in coastal mudflat soil (Figure 4A,B). This positive response reflected that more available substrates originating from sewage sludge were capable of favoring the assembly a of microbial seedbank with diverse functional traits. It was in agreement with the investigation of Pascual et al. [77], in which community level physiological profile (CLPP) analysis was used to estimate the influences of sewage sludge on soil microbial metabolic abilities of carbon sources. They observed significantly higher functional diversities in soils amended by sewage sludge as compared to unamended soils, as also described by Frac et al. [78] and Zhang et al. [79]. Interestingly, α-diversity indices of the predicted functional pathways increased with the increasing application rate of sewage sludge (Figure 4A,B). On the one hand, higher alpha diversities observed in this study may be partially due to the more diverse microbial groups introduced by the high-dose sewage sludge [80]. On the other hand, available substrate concentrations in organic amendments have been previously reported to play an important role in the functionalities of soil microbial communities [37,81]. Thus, more abundant nutrients in high-dose sewage sludge treatments might facilitate greater enrichment in bacterial populations (Figure 2 and Figure 3) associated with different functional traits. In addition, significantly distinct functional community structures were observed in soils amended by sewage sludge as compared to control soil, especially in HT treatment (Figure 4C and Table S2), which was similar to the findings of Zhang et al. [79]. The dosage-effect of sewage sludge on mudflat soil functional profiles was interpreted to be that types, quantities, availabilities, and stabilities of nutrients released from organic input decomposition differ considerably across different sludge application rates. These nutrients are capable of restructuring the soil bacterial microbiomes since they could selectively promote or inhibit specific microorganisms with different functional potential links to distinct biochemical processes [35,62,82].

The bacterial community plays important role in many soil functions such as carbon and nitrogen cycling processes [83,84,85]. In particular, microorganisms in sewage sludge have been previously reported to have significant impact on saline–sodic soils functions, especially the C- and N-related processes [86]. In this study, more diverse and abundant functional annotations of core and unique microbiomes in sewage sludge-amended soils were observed as compared to CK treatment, especially in HT treatment (Figure 4D). Specifically, functional pathways participating in carbon metabolisms (e.g., anoxygenic photoautotrophy, xylanolysis, methanol oxidation, aromatic hydrocarbon degradation, and cellulolysis, etc.) were activated as a result of sewage sludge application (Table S7). Abundant function involved in anoxygenic photoautotrophy in ST, MT, and HT treatments was likely attributed to the enriched functional genes belonging to *Chloroflexi* (Figure 2A), which has been previously reported to be capable of carbon fixation and organic material decomposition [84,87]. The activated function of carbon-containing compound degradation in sewage sludge-amended groups was in agreement with the observations of Frąc et al. [88], who demonstrated that metabolic activities of soil microbial communities in the decomposition of aromatic hydrocarbon and cellulose were enhanced induced by sewage sludge amendment. In terms of nitrogen related functions, sewage sludge treatments showed an enrichment in microbes that possess nitrate reduction, nitrogen fixation, aerobic ammonia oxidation, and nitrate denitrification potentials, etc. (Table S8 and Figure 4E), which are recognized as particularly important functional groups in soils associated with rare bacterial species [89]. Similar findings were obtained by Zhang et al. [90], who also found a rich repertoire of N-associated functionalities in a mudflat ecosystem. This result was partly attributed to the pronounced bacterial species affiliated to the phylum *Planctomycetes* (Figure 2A), which have been reported to play an important role in the biogeochemical cycle of nitrogen in mudflats [91,92]. Additionally, high concentrations of nitrogen-containing substances in sewage sludge were introduced into mudflat soil, making soil favorable for flourishment of bacterial genes involved in various nitrogen cycling processes [86]. Consequently, sewage sludge-amended soils harbored more diverse bacterial agents linked to the functionalities of carbon and nitrogen cycling processes, implying that sewage sludge amendment might be an efficient practice for functional improvement in coastal mudflat soils. However, it must be noted that FAPROTAX is generally used as a prediction tool only reflecting the metabolic and ecological functions of culturable microorganisms [45]. Therefore, more comprehensive gene-based determination methods, such as quantitative assay of functional genes and metagenomic analysis, are significant for better interpretation of the microbial community functions in response to sewage sludge management. 

### 4.4. Bacterial Community and Predicted Functional Diversities Promotion in Coastal Mudflat Soil Might Be Driven by the Microhabitat Modification Induced by Sewage Sludge Application

It is widely accepted that the soil microbial community is profoundly influenced by their ambient physicochemical properties [11,93]. Among these environmental factors, pH and salinity are increasingly considered crucial indicators reflecting the bacterial community diversity and composition, especially in saline–alkali soils [94,95,96]. Particularly, a significantly negative correlation between pH and β-diversities of bacterial was community observed in the present study (Table 3) was similar to previous research of Fang et al. [97], who also found that pH was a major factor shaping bacterial community structure in tidal marsh. One possible explanation is that most bacterial species in soil are sensitive to pH, and they prefer a relatively narrow pH spectrum [98]. Alkaline stress mitigation in mudflat soils caused by sewage sludge amendment facilitated or suppressed the enrichment of pH-dependent bacterial populations, which might play a pivotal role in forming distinct soil bacterial community structure [60]. Besides pH, salinity is one dominant driver among multiple abiotic factors in bacterial community assembly in coastal mudflat ecosystems [28,97]. In this study, significantly negative correlations were observed between soil salinity and α- and β-diversities of bacterial community, except for the richness of observed bacterial species (Table 3). Abundant literature has suggested that soils with high osmotic stress system induced by cumulative salt are generally to the disadvantage of most bacterial populations, as only a few microorganisms are broadly salt-tolerant [61,99]. Thus, sewage sludge reclaimed mudflat soil with lower salinity might favor the colonization of more divergent bacterial populations with poor salinity tolerance, which finally weakened or even replaced high-salinity tolerant species populating the pristine coastal mudflats [38]. Apart from the direct influence of soil pH and salinity on bacterial community, the two factors have been reported to be capable of affecting soil microbial communities indirectly due to their impact on the availabilities of soil nutrients (e.g., C, N, P, and S, etc.) [97,100,101]. Unsurprisingly, significantly positive correlations between contents of soil nutrients (e.g., TOC, TN, TP, AN, and AP) and most of the bacterial community diversities were observed in this study (Table 3), which were likely due to the facts that organic amendments with different nutrients (e.g., types, contents, and availabilities) drive more divergent soil bacterial microbiome formation [102,103].

In addition to soil microbial communities, functional traits linked to the microbial communities are also closely correlated with environmental factors [45,104]. Soil functionalities and their associated numerous biogeochemical processes (i.e., C- and N-related cycles) heavily rely on soil microbial communities [105,106,107]. Therefore, the dissimilar bacterial microbiomes mentioned above might imply that divergent functions related to those bacterial species will be correspondingly influenced in the ameliorated microhabitat with optimized pH and salinity [108]. In this study, the nutrients described above displayed significantly positive correlations with most predicted functional categories of carbon and nitrogen metabolisms (Figure 5). These results were in agreement with findings of Xie et al. [3], who suggested that organic amendments with abundant nutrients not only directly affect soil carbon and nitrogen cycling processes, but also had an enormous impact on microbial species and activities, which in turn affected the C- and N-relevant transformations. For instance, significantly positive relationships among nutrients and pronounced core families of *Hyphomicrobiaceae*, *Cytophagaceae*, *Pirellulaceae*, *Microbacteriaceae*, and *Phyllobacteriaceae* were observed in our study (Figure 3A and Figure 5), suggesting the enhanced carbon and nitrogen biogeochemical processes in soils after sewage sludge usage, as these families have been widely reported to be capable of participating in relevant functions [109,110,111,112]. Together, these outcomes suggested that sewage sludge significantly improved the diversity of the bacterial community and functionality mainly by ameliorating microhabitats in coastal mudflat soil, which harbored alleviated saline–alkali conditions and promoted available nutrients.

## 5. Conclusions

To the best of our knowledge, this is the first attempt to simultaneously investigate the effects of sewage sludge amendment on physicochemical characteristic, composition, diversity, and predicted function of the bacterial community in coastal mudflat soil. Results in the present study demonstrated that sewage sludge amendment not only considerably mitigated saline–alkaline and nutrient deficiency conditions, but also concurrently exerted positive effect on the improvement of community and functional diversities of bacterial microbiomes in coastal mudflat soil. Moreover, sewage sludge enabled the flourishing of bacterial core and unique microbiomes harboring more versatile carbon- and nitrogen-cycling agents. In addition, correlation analyses further revealed that modification of microhabitat condition induced by sewage sludge amendment was conducive for bacterial microbiome restructuring and functional improvement in coastal mudflat soil. Therefore, this research provided evidence that sewage sludge application can drive the reassembly of the bacterial microbiome with higher diversities and more versatile functionalities, which might exert a pivotal role during the coastal mudflat soils reclamation.

## Figures and Tables

**Table 1:** Physicochemical properties of the soil samples under different treatments.

Treatments ^§^	pH	Salinity (g·kg^−1^)	TOC (g·kg^−1^)	TN (g·kg^−1^)	TP (g·kg^−1^)	AN (mg·kg^−1^)	AP (mg·kg^−1^)
CK	8.91 ± 0.18 a	6.33 ± 1.23 a	3.96 ± 0.98 c	0.47 ± 0.01 c	0.81 ± 0.02 c	32.5 ± 1.0 c	27.2 ± 3.1 c
ST	8.59 ± 0.04 ab	4.65 ± 0.36 ab	8.80 ± 1.52 b	0.56 ± 0.04 c	1.32 ± 0.14 b	100.7 ± 10.8 b	77.5 ± 4.4 b
MT	8.53 ± 0.10 ab	4.44 ± 0.04 ab	12.11 ± 0.64 b	1.08 ± 0.02 b	1.39 ± 0.06 b	98.6 ± 14.1 b	78.8 ± 2.9 ab
HT	8.28 ± 0.10 b	3.50 ± 0.38 b	17.64 ± 1.67 a	1.38 ± 0.09 a	2.69 ± 0.13 a	163.4 ± 2.1 a	92.3 ± 6.0 a

Values (means ± standard error, n = 3) within each column followed by different letters indicate significant difference at *p* < 0.05 according to Duncan’s multiple range test. ^§^ Treatments: CK, ST, MT, and HT indicate 0, 30, 75, and 150 t ha^−1^ sewage sludge applied in mudflat saline soils, respectively.

**Table 2:** The number of unique OTUs for each treatment and overlapped OTUs for every pair of treatments.

Treatments ^§^	CK	ST	MT	HT
CK	***106*** *			
ST	*116*	***136***		
MT	*137*	*285*	***112***	
HT	*38*	*213*	*272*	***95***
Core OTUs	215	215	215	215
Total OTUs	514	718	764	634

Values in boldface type represent unique OTUs in each treatment, and italics represents overlapped OTUs between two treatments. * Only the OTUs present in all biological replicates of each treatment were selected for analyses. ^§^ Treatments: CK, ST, MT, and HT indicate 0, 30, 75, and 150 t ha^−1^ sewage sludge applied in mudflat saline soils, respectively.

**Table 3:** Spearman’s rank-order correlation coefficients between soil physicochemical characteristics and bacterial communities.

Environmental Factors	Community Composition ^#^			Community Structure ^$^
	Shannon Diversity	Richness	Evenness	PCoA1
pH				−0.76 **
Salinity	−0.64 *		−0.70 *	−0.71 **
TOC	0.71 **	0.81 **	0.70 *	0.93 **
TN	0.83 **	0.87 **	0.80 **	0.94 **
TP	0.82 **	0.76 **	0.83 **	0.86 **
AN		0.62 *		0.85 **
AP	0.58 *	0.61 *	0.64 *	0.77 **

* *p* < 0.05; ** *p* < 0.01. ^$^ PCoA1 was used to indicate microbial community structure. ^#^ Only significant correlations are exhibited.

## Data Availability

Sequences have been deposited in the NCBI under accession number PRJNA 767180.

## Supplementary Materials

The following are available online at https://www.mdpi.com/article/10.3390/biology10121302/s1. Table S1: The main physicochemical properties of mudflat soil and sewage sludge used in the present study and corresponding Chinese national standards, Table S2: PERMANOVA and PERMDISP results of bacterial communities and functional profiles among different treatments, Table S3: Pairwise comparisons of relative abundances of abundant bacterial phyla (top 10) and families (top 20) for all treatments, Table S4: The percentage of core, unique OTUs, and corresponding sequences, Table S5: Relative abundance (%) of bacterial family in core OTUs across different treatments, Table S6: Relative abundance (%) of bacterial family in unique OTUs in each treatment, Table S7: Relative abundances (%) of bacterial core OTUs-related carbon cycle relevant functions in different treatments, Table S8: Relative abundances (%) of bacterial core OTU-related nitrogen cycle relevant functions in different treatments, Table S9: Relative abundances (%) of functional annotations of unique OTUs in different treatments. Figure S1: LDA scores (A) and Taxonomic cladogram (B) for significantly different bacterial classifications between CK and sewage sludge-amended groups.
