# Supplementary material for: Structural and Predicted Functional Diversities of Bacterial Microbiome in Response to Sewage Sludge Amendment in Coastal Mudflat Soil
Source: Biology (Basel). 2021 Dec 9;10(12):1302. doi: 10.3390/biology10121302 (PMC8698727; doi:10.3390/biology10121302)
Supplement: Supplementary file 1 [file biology-10-01302-s001.zip › biology-1484921-supplementary.pdf]

Structural and Predicted Functional Diversities of Bacterial Microbiome in Response to Sewage Sludge Amendment in Coastal Mudflat Soil

Yunlong Li [1], Yimin Wang [1], Chao Shen [1], Lu Xu [1], Siqiang Yi [1], Yilin Zhao [1], Wengang Zuo [1], Chuanhui Gu [2], Yuhua Shan [1,3] and Yanchao Bai [1,3,*]

[1] *College of Environmental Science and Engineering, Yangzhou University, Yangzhou 225127, China*

[2] *Environmental Research Center, Duke Kunshan University, Kunshan, China*

[3] *Jiangsu Collaborative Innovation Center for Solid Organic Waste Resource Utilization, Nanjing 210095, China*

Running title: Sewage sludge restructures mudflat soil bacterial microbiome

* **Corresponding author:** Yanchao Bai

Address: College of Environmental Science and Engineering, Yangzhou University, Yangzhou 225127, China

E-mail: ycbai@yzu.edu.cn

Table S1. The main physicochemical properties of mudflat soil and sewage sludge used in the present study and corresponding Chinese national standards.

Characteristics	Mudflat soil	Sewage sludge	Municipal wastewater treatment plant-control standards for agricultural use in China (GB 4284-2018)
pH	9.02	6.32	5.5~8.5
Salinity (‰)	8.51	32.9	
Organic Carbon (g kg^{-1})	1.97	216.2	≥ 20
Total N (N g kg^{-1})	0.282	51.2	
Total P (P g kg^{-1})	0.507	5.51	
Alkaline N (N mg kg^{-1})	17.08	3440	
Available P (P mg kg^{-1})	6.99	813	
Total Mn (mg kg^{-1})	153.1	129.5	
Total Cd (mg kg^{-1})	1.6	3.3	15
Total Cr (mg kg^{-1})	66.4	155.7	1000
Total Cu (mg kg^{-1})	15.9	1121.9	1500
Total Ni (mg kg^{-1})	30.9	52.8	200
Total Zn (mg kg^{-1})	56.2	2127.3	3000

Table S2. PERMANOVA and PERMDISP results of bacterial communities and functional profiles among different treatments.

Bacterial community								Functional categories							
**Source**	**PERMANOVA**					**Pairwise tests**		**Source**	**PERMANOVA**					**Pairwise tests**	
	**df**	**SS**	**MS**	**Pseudo-*F***	***P***	***P***			**df**	**SS**	**MS**	**Pseudo-*F***	***P***	***P***	
Treatment	3	1.788	0.596	23.019	***	CK, ST	0.125	Treatment	3	1.788	0.596	23.019	***	CK, ST	0.327
Residuals	8	0.207	0.026			CK, MT	0.216	Residuals	8	0.207	0.026			CK, MT	0.40
Total	11	1.995				CK, HT	0.031	Total	11	1.995				CK, HT	0.021
						ST, MT	0.125							ST, MT	0.173
						ST, HT	0.163							ST, HT	0.167
						MT, HT	0.206							MT, HT	0.167
	**PERMDISP**					**Pairwise tests**			**PERMDISP**					**Pairwise tests**	
**Source**	**df**	**SS**	**MS**	**Pseudo-*F***	***P***	***P***		**Source**	**df**	**SS**	**MS**	**Pseudo-*F***	***P***	***P***	
Treatment	3	0.010	0.003	0.375	0.793	CK, ST	0.788	Treatment	3	0.006	0.002	1.542	0.277	CK, ST	0.611
Residuals	8	0.070	0.009			CK, MT	0.453	Residuals	8	0.011	0.001			CK, MT	0.100
						CK, HT	0.835							CK, HT	0.050
						ST, MT	0.412							ST, MT	0.497
						ST, HT	0.251							ST, HT	0.387
						MT, HT	0.291							MT, HT	0.582

SS, sum of squares; MS, means of squares; "***" indicate $P < 0.001$; number of permutations: 999.

Table S3. Relative abundances (%) of abundant bacterial phyla (top 10) and families (top 20) for all treatments.

TAXA		Treatments §			
		CK	ST	MT	HT
Phyla	*Bacteroidetes*	45.89 ± 6.13 a	48.93 ± 4.82 a	41.10 ± 1.91 a	45.89 ± 3.82 a
	Proteobacteria	34.92 ± 6.22 a	22.74 ± 1.36 a	20.84 ± 0.97 a	23.78 ± 0.85 a
	Chloroflexi	5.13 ± 0.49 a	9.47 ± 2.22 a	11.87 ± 1.05 a	8.22 ± 1.50 a
	Actinobacteria	5.02 ± 0.46 a	4.59 ± 1.18 a	6.88 ± 0.97 a	6.89 ± 1.68 a
	Acidobacteria	5.23 ± 0.41 b	3.64 ± 0.05 c	10.11 ± 0.76 a	2.07 ± 0.24 d
	Planctomycetes	1.55 ± 0.04 c	4.83 ± 0.53 ab	3.96 ± 0.25 b	6.01 ± 0.55 a
	Firmicutes	1.01 ± 0.03 a	1.83 ± 0.08 a	1.72 ± 0.01 a	3.88 ± 1.23 a
	Gemmatimonadetes	0.19 ± 0.04 c	0.53 ± 0.07 b	0.81 ± 0.01 a	0.64 ± 0.07 ab
	Nitrospirae	0.22 ± 0.02 b	0.44 ± 0.04 a	0.22 ±0.01 b	0.20 ± 0.02 b
	Chlorobi	0.02 ± 0.01 d	0.54 ± 0.06 a	0.34 ± 0.02 b	0.14 ± 0.02 c
	Others	0.82 ± 0.10 b	2.47 ± 0.53 a	2.16 ± 0.09 a	2.29 ± 0.18 a
Family	*Flavobacteriaceae*	37.70 ± 6.26 a	36.15 ± 3.09 a	39.53 ± 4.60 a	31.56 ± 1.33 a
	Xanthomonadaceae	21.93 ± 5.44 a	7.59 ± 0.50 b	4.02 ± 0.68 b	4.91 ± 0.49 b
	Hyphomicrobiaceae	1.41 ± 0.10 d	6.18 ± 0.17 a	2.74 ± 0.21 c	4.74 ± 0.19 b
	Cytophagaceae	0.64 ± 0.08 d	4.35 ± 0.87 b	6.21 ± 0.18 a	2.62 ± 0.28 c
	Pirellulaceae	1.06 ± 0.06 c	3.77 ± 0.44 a	3.14 ± 0.35 ab	2.60 ± 0.12 b
	Saprospiraceae	6.80 ± 0.44 a	0.49 ± 0.06 c	0.45 ± 0.05 c	1.80 ± 0.12 b
	Micrococcaceae	2.18 ± 0.27 a	1.26 ± 0.32 a	0.61 ± 0.04 a	1.65 ± 0.24 a
	Comamonadaceae	2.49 ± 0.61 a	0.29 ± 0.03 b	0.75 ± 0.22 b	0.73 ± 0.00 b
	Planctomycetaceae	0.24 ± 0.02 b	1.67 ± 0.16 a	1.20 ± 0.17 a	0.97 ± 0.11 a
	Chitinophagaceae	0.43 ± 0.06 a	1.67 ± 0.19 a	0.79 ± 0.19 a	1.01 ± 0.05 a
	Alteromonadaceae	0.15 ± 0.02 b	0.37 ± 0.03 b	2.64 ± 0.84 a	0.38 ± 0.02 b
	Peptostreptococcaceae	0.02 ± 0.01 b	1.43 ± 0.26 a	0.65 ± 0.06 a	0.85 ± 0.02 a
	Geobacteraceae	1.23 ± 0.08 a	0.24 ± 0.02 b	0.88 ± 0.19 a	0.53 ± 0.02 a
	Erythrobacteraceae	0.79 ± 0.12 b	0.29 ± 0.04 c	1.27 ± 0.38 a	0.36 ± 0.05 c
	Caldilineaceae	0.41 ± 0.05 b	0.36 ± 0.03 b	0.47 ± 0.06 b	1.44 ± 0.05 a
	Microbacteriaceae	0.08 ± 0.01 c	1.58 ± 0.45 a	0.30 ± 0.05 c	0.68 ± 0.10 b
	Nocardioidaceae	0.89 ± 0.06 a	0.65 ± 0.16 a	0.50 ± 0.14 a	0.45 ± 0.06 a
	Cyclobacteriaceae	0.02 ± 0.01 b	0.98 ± 0.10 a	0.82 ± 0.27 a	0.39 ± 0.06 b
	Caulobacteraceae	0.87 ± 0.12 a	0.62 ± 0.09 a	0.20 ± 0.02 b	0.34 ± 0.03 b
	Sinobacteraceae	0.23 ± 0.01 a	0.48 ± 0.07 a	0.43 ± 0.08 a	0.83 ± 0.02 a
	Others	20.43 ± 0.30 c	29.57 ± 3.30 bc	32.40 ±4.73 ab	41.18 ± 0.86 a

§ Treatments: CK, ST, MT, and HT indicate 0, 30, 75, and 150 t ha^{-1} sewage sludge applied in mudflat saline soils, respectively. Values (means ± standard error, n = 3) within each raw followed by different letters indicate significant difference at $P < 0.05$ according to Duncan's multiple range test.

Table S4. The percentage of core, unique OTUs, and corresponding sequences.

Treatments §	Core (%)		Unique (%)	
	OTUs	Sequences	OTUs	Sequences
CK	41.8	85.4	20.6	2.2
ST	29.9	72.8	18.9	2.8
MT	28.1	66.1	14.7	1.6
HT	33.9	48.5	15.0	1.5

§ Treatments: CK, ST, MT, and HT indicate 0, 30, 75, and 150 t ha-1 sewage sludge applied in mudflat saline soils, respectively.

Table S5. Relative abundance (%) of bacterial family in core OTUs across different treatments.

Core families	Treatments §			
	CK	ST	MT	HT
Flavobacteriaceae	43.59 ± 7.24 a	47.43 ± 4.99 a	34.31 ± 1.61 ab	20.17 ± 1.34 c
Xanthomonadaceae	25.63 ± 6.34 a	5.36 ± 0.67 b	6.79 ± 0.6 b	11.00 ± 0.11 b
Hyphomicrobiaceae	1.57 ± 0.12 d	3.64 ± 0.20 c	7.04 ± 0.23 b	12.69 ± 0.51 a
Cytophagaceae	0.63 ± 0.07 c	5.25 ± 0.21 ab	2.97 ± 0.36 bc	6.95 ± 1.77 a
Pirellulaceae	0.86 ± 0.04 c	2.89 ± 0.43 b	2.78 ± 0.14 b	6.11 ± 0.90 a
Micrococcaceae	2.58 ± 0.31 a	0.86 ± 0.07 b	2.52 ± 0.37 a	2.64 ± 0.65 a
Saprospiraceae	4.03 ± 0.32 a	0.32 ± 0.07 c	1.43 ± 0.11 b	0.55 ± 0.11 bc
Comamonadaceae	2.81 ± 0.68 a	1.01 ± 0.24 b	1.06 ± 0.01 b	0.52 ± 0.07 b
Alteromonadaceae	0.15 ± 0.03 b	3.52 ± 1.21 a	0.48 ± 0.05 b	0.72 ± 0.05 b
Microbacteriaceae	0.09 ± 0.01 b	0.30 ± 0.07 b	0.91 ± 0.14 b	2.72 ± 0.77 a
Nocardioidaceae	0.97 ± 0.07 a	0.70 ± 0.21 a	0.75 ± 0.10 a	1.35 ± 0.24 a
Geobacteraceae	1.35 ± 0.09 a	1.16 ± 0.27 ab	0.70 ± 0.01 ab	0.50 ± 0.04 b
Erythrobacteraceae	0.69 ± 0.12 ab	1.59 ± 0.51 a	0.44 ± 0.04 c	0.52 ± 0.06 bc
Caulobacteraceae	0.95 ± 0.15 b	0.26 ± 0.03 b	0.38 ± 0.01 a	1.18 ± 0.13 b
Caldilineaceae	0.11 ± 0.02 b	0.30 ± 0.04 b	1.60 ± 0.07 a	0.50 ± 0.05 b
Phyllobacteriaceae	0.13 ± 0.02 b	0.43 ± 0.06 b	0.39 ± 0.03 b	1.21 ± 0.18 a
Others	6.56 ± 0.22 c	11.35 ± 1.05 b	21.38 ± 0.83 a	12.92 ± 0.47 b
Unclassified	7.30 ± 0.45 a	13.62 ± 3.36 a	14.07 ± 1.53 a	17.74 ± 2.99 a

§ Treatments: CK, ST, MT, and HT indicate 0, 30, 75, and 150 t ha-1 sewage sludge applied in mudflat saline soils, respectively.

Table S6. Relative abundance (%) of bacterial family in unique OTUs in each treatment.

Unique families	Treatments §			
	CK	ST	MT	HT
Anaerolinaceae	ND	ND	9.97	ND
Caldilineaceae	1.77	ND	ND	ND
Chitinophagaceae	6.00	ND	ND	ND
Clostridiaceae	ND	1.93	ND	ND
Comamonadaceae	1.64	ND	ND	ND
Coxiellaceae	ND	8.87	1.84	3.24
Cryomorphaceae	ND	ND	ND	6.47
Cytophagaceae	1.23	3.29	1.56	2.91
Flavobacteriaceae	ND	3.97	ND	ND
Ignavibacteriaceae	ND	1.89	ND	ND
Isosphaeraceae	ND	ND	ND	4.54
Legionellaceae	ND	1.86	ND	ND
Pirellulaceae	1.38	4.30	2.06	4.00
Planctomycetaceae	2.54	4.71	1.84	8.21
Rhodobacteraceae	ND	ND	2.05	ND
Saprospiraceae	2.29	ND	6.40	ND
Sinobacteraceae	ND	ND	ND	4.75
Sphingomonadaceae	ND	ND	ND	3.03
Syntrophomonadaceae	ND	ND	1.54	ND
Xanthomonadaceae	5.98	ND	ND	ND
Others	12.88	7.62	16.54	5.29
Unclassified	53.02	43.17	42.51	30.67

§ Treatments: CK, ST, MT, and HT indicate 0, 30, 75, and 150 t ha^{-1} sewage sludge applied in mudflat saline soils, respectively. ND indicates not detected.

Table S7. Relative abundances (%) of bacterial core OTUs-related carbon cycle relevant functions in different treatments.

Functions	Treatments §			
	CK	ST	MT	HT
anoxygenic photoautotrophy S oxidizing	0.37 ± 0.07 d	0.84 ± 0.12 c	1.80 ± 0.09 b	3.36 ± 0.02 a
anoxygenic photoautotrophy	0.37 ± 0.07 d	0.84 ± 0.12 c	1.80 ± 0.09 b	3.36 ± 0.02 a
photoautotrophy	0.37 ± 0.07 d	0.84 ± 0.12 c	1.80 ± 0.09 b	3.36 ± 0.02 a
photoheterotrophy	0.37 ± 0.07 d	0.84 ± 0.12 c	1.80 ± 0.09 b	3.36 ± 0.02 a
phototrophy	0.37 ± 0.07 d	0.84 ± 0.12 c	1.80 ± 0.09 b	3.36 ± 0.02 a
xylanolysis	0.80 ± 0.07 b	2.45 ± 0.85 a	0.43 ± 0.04 b	0.54 ± 0.02 b
aromatic compound degradation	0.14 ± 0.02 b	2.32 ± 0.82 a	0.46 ± 0.03 b	0.55 ± 0.03 b
cellulolysis	0.11 ± 0. 00 b	2.27 ± 0.80 a	0.34 ± 0.03 b	0.44 ± 0.03 b
methanol oxidation	0.01 ± 0.00 c	0.03 ± 0.00 c	0.30 ± 0.02 a	0.16 ± 0.05 b
methylotrophy	0.01 ± 0.00 c	0.03 ± 0.00 c	0.30 ± 0.02 a	0.16 ± 0.01 b
aromatic hydrocarbon degradation	0.01 ± 0.00 b	0.02 ± 0.01 b	0.07 ± 0.01 a	0.06 ± 0.01 a
aliphatic non methane hydrocarbon degradation	0.01 ± 0.00 b	0.02 ± 0.01 b	0.07 ± 0.01 a	0.06 ± 0.01 a
hydrocarbon degradation	0.01 ± 0.00 b	0.02 ± 0.01 b	0.07 ± 0.01 a	0.06 ± 0.01 a
Accumulative relative abundance (%)	2.96 ± 0.36 c	11.33 ± 1.90 b	11.04 ± 0.58 b	18.86 ± 0.03 a

Values (means ± standard error, n = 3) within each raw followed by different letters represent significant difference at $P < 0.05$ according to Duncan's multiple range test.

§ Treatments: CK, ST, MT, and HT indicate 0, 30, 75, and 150 t ha^{-1} sewage sludge applied in mudflat saline soils, respectively.

Table S8. Relative abundances (%) of bacterial core OTUs-related nitrogen cycle relevant functions in different treatments.

Functions	Treatments §			
	CK	ST	MT	HT
nitrate reduction	0.60 ± 0.06 d	1.09 ± 0.08 c	2.25 ± 0.10 b	3.81 ± 0.12 a
nitrate respiration	0.52 ± 0.07 d	1.07 ± 0.09 c	2.23 ± 0.10 b	3.78 ± 0.12 a
nitrogen respiration	0.52 ± 0.07 d	1.07 ± 0.09 c	2.23 ± 0.10 b	3.78 ± 0.12 a
nitrite respiration	0.40 ± 0.07 d	0.89 ± 0.11 c	2.12 ± 0.11 b	3.55 ± 0.03 a
nitrate denitrification	0.38 ± 0.07 d	0.87 ± 0.12 c	2.10 ± 0.11 b	3.53 ± 0.03 a
nitrite denitrification	0.38 ± 0.07 d	0.87 ± 0.12 c	2.10 ± 0.11 b	3.53 ± 0.03 a
nitrous oxide denitrification	0.38 ± 0.07 d	0.87 ± 0.12 c	2.10 ± 0.11 b	3.53 ± 0.03 a
denitrification	0.38 ± 0.07 d	0.87 ± 0.12 c	2.10 ± 0.11 b	3.53 ± 0.03 a
nitrification	0.24 ± 0.03 c	0.46 ± 0.04 a	0.30 ± 0.01 bc	0.35 ± 0.03 b
aerobic nitrite oxidation	0.17 ± 0.02 c	0.37 ± 0.03 a	0.21 ± 0.01 bc	0.24 ± 0.02 b
aerobic ammonia oxidation	0.07 ± 0.02 a	0.09 ± 0.02 a	0.09 ± 0.00 a	0.11 ± 0.01 a
ureolysis	0.01 ± 0.00 b	0.04 ± 0.01 b	0.09 ± 0.01 b	0.27 ± 0.01 a
nitrogen fixation	0.09 ± 0.01 a	0.09 ± 0.03 a	0.07 ± 0.01 a	0.07 ± 0.00 a
nitrate ammonification	0.02 ± 0.01 a	0.02 ± 0.01 a	0.02 ± 0.01 a	0.02 ± 0.00 a
nitrite ammonification	0.02 ± 0.01 a	0.02 ± 0.01 a	0.02 ± 0.01 a	0.02 ± 0.00 a
Accumulative relative abundance (%)	4.18 ± 0.54 d	8.70 ± 0.77 c	18.05 ± 0.85 b	30.08 ± 0.52 a

Values (means ± standard error, *n* = 3) within each raw followed by different letters represent significant difference at $P < 0.05$ according to Duncan's multiple range test.

§ Treatments: CK, ST, MT, and HT indicate 0, 30, 75, and 150 t ha^{-1} sewage sludge applied in mudflat saline soils, respectively.

Table S9. Relative abundances (%) of functional annotations of unique OTUs in different treatments.

Functions	Treatments §			
	CK	ST	MT	HT
aerobic ammonia oxidation	ND	ND	6.75	ND
animal parasites or symbionts	6.15	0.58	5.24	ND
anoxygenic photoautotrophy	ND	1.19	3.51	ND
anoxygenic photoautotrophy S oxidizing	ND	1.19	3.51	ND
cellulolysis	ND	ND	ND	3.94
cyanobacteria	1.28	7.83	ND	1.51
denitrification	ND	ND	ND	1.46
fermentation	3.3	1.41	2.72	3.55
human gut	ND	0.58	2.72	ND
human pathogens all	6.15	ND	2.52	ND
hydrocarbon degradation	2.03	ND	2.26	ND
intracellular parasites	2.14	37.95	27.84	24.78
mammal gut	ND	0.58	2.72	ND
methanotrophy	2.03	ND	ND	ND
methylotrophy	2.03	ND	ND	ND
nitrate denitrification	ND	ND	ND	1.46
nitrate reduction	7.04	ND	ND	1.46
nitrate respiration	5.26	ND	ND	1.46
nitrification	ND	ND	6.75	ND
nitrite denitrification	ND	ND	ND	1.46
nitrite respiration	ND	ND	ND	1.46
nitrogen fixation	ND	0.58	ND	ND
nitrogen respiration	5.26	ND	ND	1.46
nitrous oxide denitrification	ND	ND	ND	1.46
oxygenic photoautotrophy	1.28	7.83	ND	1.51
photoautotrophy	1.28	9.02	3.51	1.51
photoheterotrophy	ND	ND	1.41	ND
phototrophy	1.28	9.02	4.92	1.51
respiration of sulfur compounds	ND	1.08	ND	ND
sulfate respiration	ND	1.08	ND	ND
ureolysis	ND	ND	4.94	ND

§ Treatments: CK, ST, MT, and HT indicate 0, 30, 75, and 150 t ha^{-1} sewage sludge applied in mudflat saline soils, respectively. ND indicates not detected.

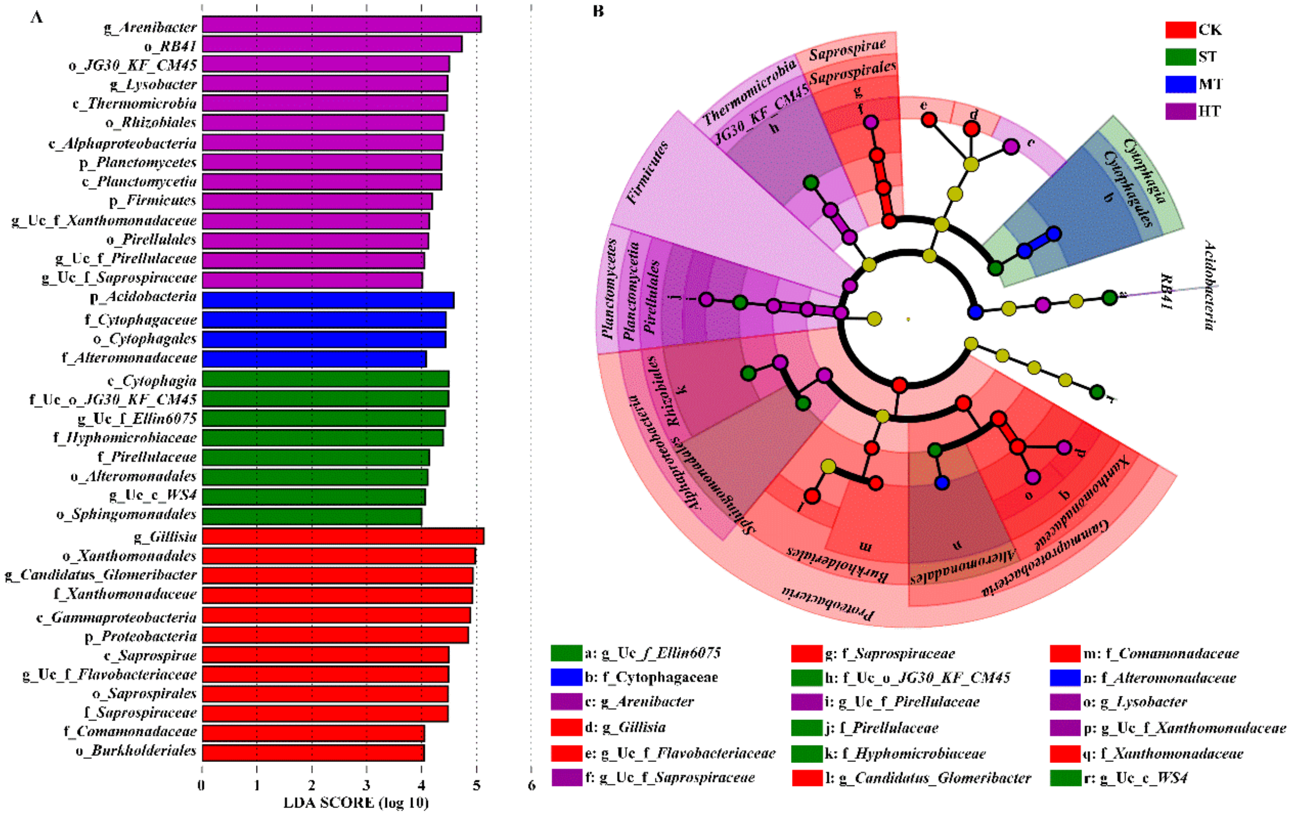

Figure S1. LDA scores (A) and Taxonomic cladogram (B) for significantly different bacterial classifications between CK and sewage sludge amended groups (Kruskal-Wallis test, $P < 0.05$, LDA > 4 (log 10)). The letters in front of the taxonomic designations indicate the taxonomic levels, i.e., p, phylum; c, class; o, order; f, family; g, genus. “Uc_” means unidentified, and letters followed by “Uc_” represent the most detailed classification. Treatments: CK, ST, MT, and HT indicate 0, 30, 75, and 150 t ha-1 sewage sludge applied in mudflat saline soils, respectively.
